# Clinical control in COPD and therapeutic implications: The EPOCONSUL audit

**DOI:** 10.1371/journal.pone.0314299

**Published:** 2025-01-09

**Authors:** Myriam Calle Rubio, Marc Miravitlles, Juan José Soler Cataluña, José Luis López-Campos, Bernardino Alcázar Navarrete, Manuel E. Fuentes Ferrer, Juan Luis Rodríguez Hermosa

**Affiliations:** 1 Pulmonology Department, Department of Medicine, Hospital Clínico San Carlos, School of Medicine, Instituto de Investigación Sanitaria del Hospital Clínico San Carlos (IdISSC), CIBER de Enfermedades Respiratorias (CIBERES), Universidad Complutense de Madrid, Madrid, Spain; 2 Pulmonology Department, Hospital Universitari Vall d’Hebron, Vall d’Hebron Institut de Recerca (VHIR), Vall d’Hebron Barcelona Hospital Campus, CIBER de Enfermedades Respiratorias (CIBERES), Barcelona, Spain; 3 Pulmonology Department, Hospital Arnau de Vilanova-Lliria, Valencia, Spain; 4 Respiratory Disease Medical-Surgical Unit, Instituto de Biomedicina de Sevilla (IBiS), Hospital Universitario Virgen del Rocío/Universidad de Sevilla, Sevilla, Spain; 5 Pulmonology Department, Hospital Virgen de las Nieves, Granada, Spain; 6 Unidad de Investigación, Hospital Universitario Nuestra Señora de Candelaria, Santa Cruz de Tenerife, Spain; 7 Medicine Department, CIBER de Enfermedades Respiratorias (CIBERES), Instituto de Salud Carlos III, Universitat de València, Valencia, Madrid; 8 CIBER de Enfermedades Respiratorias (CIBERES), Instituto de Salud Carlos III, Madrid, Spain; 9 IBS-Granada, Medicine Department, Universidad de Granada, Granada, Spain; National and Kapodistrian University of Athens, GREECE

## Abstract

**Objective:**

This study aimed to evaluate clinical control in chronic obstructive pulmonary disease (COPD), the consequences in terms of treatment decisions, and their potentially associated factors during follow-up of patients in real-life clinical practice.

**Methods:**

EPOCONSUL 2021 is a cross-sectional audit that evaluated the outpatient care provided to patients with a diagnosis of COPD in respiratory clinics in Spain and multivariable logistic regression models to assess the relationships between clinical control and clinical inertia.

**Results:**

4225 patients from 45 hospitals in Spain were audited. Clinical control was analyzed in 1804 (42.7%) patients who met all the Spanish COPD Guidelines (GesEPOC) criteria. 49.1% of patients were classified as uncontrolled, and 42.2% of patients disagreed with the level of control determined by their doctor, which was reported as good during the visit. There was therapeutic inertia (TI), in other words not making any change or taking any action in the treatment of COPD, in 68.4% of uncontrolled patients and no action was taken during the visit for 9.1% of uncontrolled patients. Factors associated with TI in uncontrolled patients were disagreement with the degree of control reported by the doctor who performed the examination ☯physician classifies and reports disease as controlled versus uncontrolled, OR: 3.37 (2.33–4.88), p<0.001] and having a lower burden of associated comorbidities ☯Charlson comorbidity index ≥3 versus <3, OR 0.8 (0.1–3.0), p = 0.014]. The probability of disagreeing with the physician’s classification of the degree of COPD control in uncontrolled patients was lower in patients with severe exacerbations ☯OR 0.3 (0.17–0.78), p = 0.009] and those with more exacerbations in the last year ☯OR 0.6 (0.4–0.9), p = 0.019].

**Conclusions:**

Therapeutic inertia exists in more than half of uncontrolled patients and is more likely when there is disagreement with the assessment of the physician responsible for the visit, who reported there being good disease control, a situation that was more likely in patients with less history of exacerbations.

## Introduction

Clinical practice guidelines in COPD establish the reduction of symptoms and minimization of risk as the main therapeutic objectives [1, 2]. These objectives make it necessary to adapt actions to the changes experienced by patients throughout their evolution, considering therapeutic success to be the achievement of disease control. Thus, COPD clinical control is a measure proposed as a tool to help clinicians make decisions during the visit [1, 3]. Previous studies have shown that COPD control status is predictive of future exacerbations and time until the next exacerbation [4–6], as well as providing relevant information on health status [5] and survival prognosis [7], thus helping to identify patients that require clinician action.

Different studies indicate that there is a gap between the healthcare that patients receive and the guideline recommendations in the process of COPD care [8, 9], although adherence to clinical guidelines is a predictor of a favorable outcome [10]. These gaps often need to be considered in the context of clinical inertia, which is defined as "recognition of the problem but lack of action" [11]. Clinical inertia is a broad concept, encompassing physician, patient and health system factors including a failure to assess risk, screen for and manage risk factors and complications, address nonpharmacological factors and refer appropriately. Therapeutic inertia (TI) is one component of clinical inertia, which is when providers fail to initiate medications or intensify treatment when treatment goals are not met [12]. It has been extensively studied in diabetes [13] and hypertension [14] and it has been reported to potentially account for 80% of cardiovascular events, suggesting that it may be an appropriate focus for quality improvement. In contrast, clinical inertia related to the management of COPD has been less commonly researched, and little is known about physicians’ practices and why physicians do not initiate medication or do not intensify treatment if therapeutic goals are not reached [15].

COPD is a disease characterized by frequent decompensations, which are responsible for increased morbidity and mortality. These constitute one of the main reasons for the adjustment and personalization of treatment in patient follow-up. Therefore, overcoming clinical inertia is crucial in the management of patients with COPD. Consequently, it is necessary to assess the main elements on which physicians base their therapeutic decisions.

Our analysis aimed to evaluate clinical control in COPD, the consequences in terms of treatment decisions and their potentially associated factors during the follow-up of patients in real-life clinical practice.

## Methodology

The methodology of the EPOCONSUL audit has been previously reported [16]. Briefly, the EPOCONSUL audit promoted by the Spanish Society of Pneumology and Thoracic Surgery (SEPAR) was designed to evaluate outpatient care provided to patients with COPD in respiratory clinics in Spain as an observational non-interventional cross-sectional study.

SEPAR sent an official invitation to participate in the study to all the Spanish respiratory units with outpatient respiratory clinics according to the Ministry of Health registry and the SEPAR member registry. The study inclusion was performed between April 15, 2021, and January 31, 2022. Recruitment was intermittent; every month, each investigator recruited the clinical records of the first 10 patients identified as being diagnosed with COPD who were seen in the outpatient respiratory clinic. Subsequently, the patients identified were reevaluated to determine if they met the inclusion/exclusion criteria (aged ≥40 years, smokers or ex-smokers with COPD diagnosed on the basis of spirometric tests and with previous follow-up for at least 1 year in a respiratory outpatient clinic as described in S1 Appendix. The level of risk was defined according to GesEPOC criteria (post-bronchodilator FEV1%, degree of dyspnea and history of exacerbations) described in S2 Appendix. The level of clinical control of COPD was defined by post hoc analysis of data collected at the last audited visit according to the criteria established by GesEPOC based on two components: impact and stability, described in S3 Appendix. The degree of COPD control calculated and recorded at the visit by the doctor responsible was assessed. Clinical inertia was defined as not taking any action during the visit, requesting a test or making a change in the treatment for an uncontrolled patient and TI was defined as not making any change or taking action in the treatment of COPD during the visit in an uncontrolled patient. Investigators participating in 2021 EPOCONSUL are included in S4 Appendix.

The protocol was approved by the Ethics Committee of the Hospital Clínico San Carlos (Madrid, Spain; internal code 20/722-E). Additionally, according to current research laws in Spain, the ethics committee at each participating hospital evaluated and agreed to the study protocol. The need for informed consent was waived because ours is a clinical audit, in addition to the non-interventional nature of the study, the anonymization of data and the blind evaluation of clinical performance. The medical staff responsible for the outpatient respiratory clinic weren’t informed about the audit in order to avoid modifications to the usual clinical practice and to preserve the blinding of the clinical performance evaluation.

### Statistical analysis

Qualitative variables were summarized as frequency distribution and continuous variables as mean values and standard deviations (SD). Continuous, non-normally distributed variables were expressed as medians and interquartile ranges (IQR). The association between qualitative variables was performed using the chi-square test, the comparison of means between quantitative variables and binary outcome variables was assessed using the Student’s T-test, and the non-parametric Mann-Whitney U test was used in the case of continuous non-normally distributed variables.

Four multivariable logistic regression models, using cluster-robust standard errors to take into account patients tested within the same hospital, were fitted in order to identify factors associated with clinically uncontrolled COPD, the physician’s determination of good clinical control, clinical inertia and therapeutic inertia in uncontrolled patients. Adjusted odds ratios (OR) and their 95% confidence intervals are shown. Independent variables with a p-value of p<0.05 in the univariate analysis and/or those considered clinically relevant were added to each of the models. Statistical significance was assumed as p<0.05. All analyses were performed using Stata software version 16 (StataCorp LLC, College Station, TX, USA).

## Results

### Population of the study

**Fig 1:**
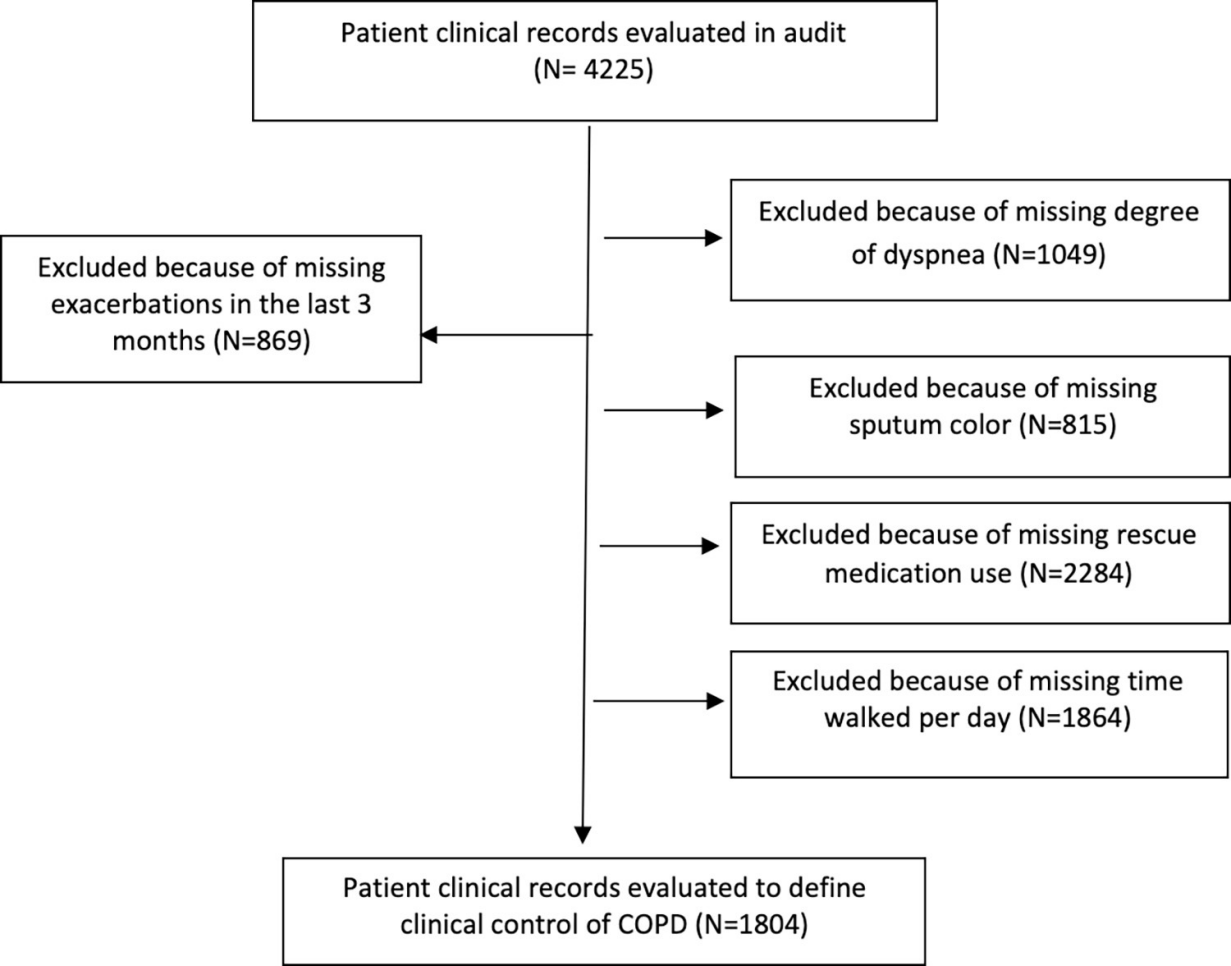
STROBE flow chart of the sampling process. A patient may have a missing value in more than one criterion.

A total of 4225 patients diagnosed with COPD at 45 centers were audited. Of these, 1804 (42.7%) patients who met all the GesEPOC criteria recorded at the visit were analyzed to define the level of clinical impact and stability in order to assess the level of clinical control of COPD. The sampling process is shown in Fig 1.

### Clinical control of COPD and factors associated with control in COPD

**Fig 2:**
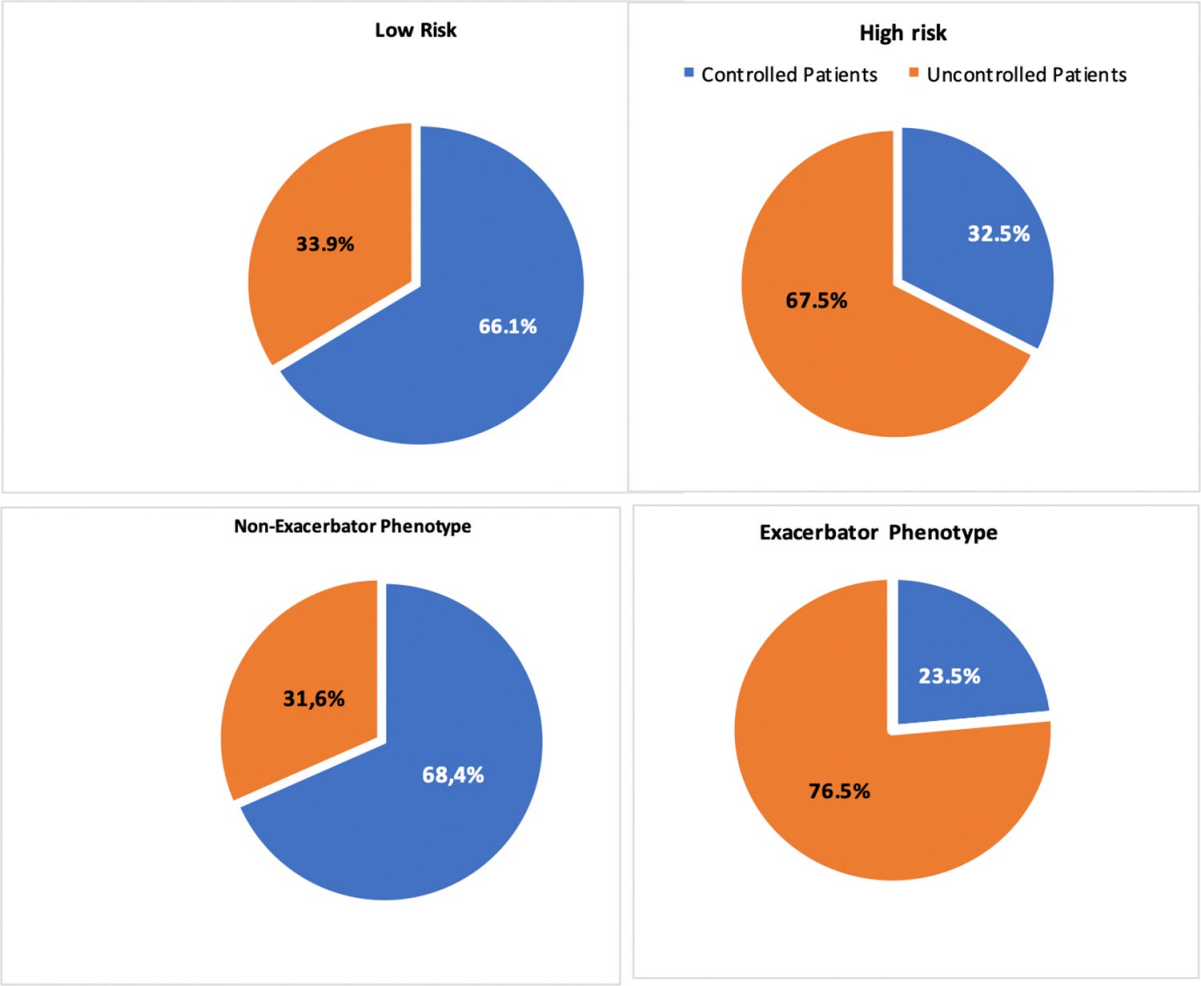
Distribution of clinical control of COPD according to risk level and phenotype GesEPOC. Data are represented as percentages.

1288 (71.4%) were men, and the mean age was 69.7 (9.0) years. The mean forced expiratory volume first second (FEV1) (%) was 52.3 (18.2). COPD was uncontrolled in 49.1% of patients and controlled in 50.9% according to the level of clinical control by GesEPOC criteria. Clinical characteristics according to clinical control are shown in Table 1. Fig 2A shows how they are distributed according to risk level and clinical phenotype based on GesEPOC criteria. The following factors were independently associated with uncontrolled COPD (Table 2): depression (OR 1.7, 95% CI 1.2–2.5; p = 0.002), chronic bronchial infection (OR 1.4, 95% CI 1.1–2.1; p = 0.043), use of long-term oxygen therapy (OR 2.4, 95% CI 1.7–3.3; p<0.001) and being cared for in a tertiary hospital center (OR 1.8, 95% CI1.2–2.9; p = 0.006).

**Table 1:** Characteristics according to level of clinical control of COPD.

	Total N = 1804	Controlled Patients N = 919	Uncontrolled Patients N = 885	p
	**Clinical Characteristics**			
Gender (male), n (%)	1288 (71.4)	660 (71.8)	628 (71)	0.363
Age (years), m (SD)	69.7(9.0)	68.4 (8.9)	71.1 (9.0)	<0.001
Current smokers, %	491 (27.2)	261 (28.4)	230 (26)	0.136
Pack-years, m (SD)	49.1 (23.4)	45.3 (22.4)	53.0 (23.8)	<0.001
BMI kg/m2, m (SD)	27.5 (5.4)	27.4 (5.4)	27.5 (5.5)	0.881
Charlson index, median, (IQR)	1 (1–3)	1 (1–2)	2 (1–3)	<0.001
Charlson index ≥3, n (%)	508 (28.9)	219 (23.9)	289 (32.7)	<0.001
Myocardial infarction, n (%)	203 (11.3)	79 (8.6)	124 (14)	<0.001
Congestive heart failure, n (%)	136 (7.5)	33 (3.6)	103 (11.6)	<0.001
Diabetes, n (%)	342 (19)	141 (15.3)	201 (22.7)	<0.001
Neoplasm, n (%)	278 (16.2)	94 (10.2)	124 (14)	0.014
Asthma, n (%)	119 (6.6)	61 (6.6)	58 (6.6)	0.509
Obstructive apnea syndrome, n (%)	390 (21.6)	171 (18.6)	219 (24.7)	0.001
Depression, n (%)	249 (13.8)	82 (8.9)	167 (18.9)	<0.001
Anxiety, n (%)	242 813.4)	107 (11.6)	135 (15.3)	0.015
Dyspnea (mMRC) ≥2, %	995 (56)	250 (27.8)	745 (84.9)	<0.001
CAT questionnaire >10, n (%)	547 (70.5)	209 (54.7)	338 (85.8)	<0.001
Chronic bronchitis criteria, n (%)	717 (39.7)	280 (30.5)	437 (49.4)	<0.001
Chronic colonization, n (%)	255 (14.1)	87 (9.5)	168 (19)	<0.001
Symptoms suggestive of asthma, n (%)	219 (12.1)	114 (12.9)	105 (11.4)	0.344
Post-FEV_1_, % predicted, m (SD)	52.3 (18.2)	56.2 (18.8)	48.2 (16.6)	<0.001
KCO, % predicted, m (SD)	66.3 (23.1)	69.5 (23.1)	63.0 (22.7)	<0.001
Number of exacerbations in last year, median, (IQR)	0 (0–1)	0 (0–1)	1 (0–2)	<0.001
≥1 hospital admissions in the last year, n (%)	397 (22)	71 (7.7)	326 (39.4)	<0.001
BODE value, median, (IQR)	4 (2–6)	3 (1–4)	5 (3–6)	<0.001
BODEx value, median, (IQR)	3 (1–5)	2 (0–3.7)	4 (3–5)	<0.001
GOLD group, n (%)				<0.001
• A	257 (29.7)	217 (52)	40 (8.9)	
• B	240 (27.7)	100 (24)	140 (31.2)	
• E	369 (42.6)	100 (24)	269 (59.1)	
GesEPOC high-risk level, n (%)	813 (65.9)	264 (45.1)	549 (84.6)	<0.001
GesEPOC phenotype, n (%)				<0.001
• Non-exacerbator	676 (47.4)	462 (67.6)	214 (28.8)	
• Exacerbator with chronic bronchitis	267 (18.7)	54 (7.9)	213 (28.7)	
• Exacerbator with emphysema	315 (22.1)	83 (12.2)	232 (31.2)	
• Asthma-COPD	168 (11.8)	84 (12.3)	84 (11.3)	
• Monotherapy (LAMA), n (%)	112 (6.3)	96 (10.6)	16 (1.8)	0.039
• Monotherapy (LABA), n (%)	8 (0.5)	6 (0.7)	2 (0.2)	
• LAMA+LABA combination, n (%)	605 (34.1)	361 (39.9)	244 (27.8)	
• LABA+ICS combination, n (%)	135 (7.6)	82 (9.1)	53 (6)	
• Triple therapy (LAMA+LABA+ICS), n (%)	916 (51.6)	356 (39.3)	560 (63.8)	
Any change in current medication advised, n (%)	467 (25.9)	187 (20.3)	280 (31.6)	<0.001
Long-term oxygen therapy, n (%)	463 (25.7)	108 (11.8)	355 (40.1)	<0.001
Home ventilation, n (%)	149 (8.3)	45 (4.9)	104 (11.8)	<0.001
Respiratory rehabilitation, n (%)	265 (14.7)	98 (10.7)	167 (18.9)	<0.001
	**Care process**			
Level of complexity of hospital				<0.001
Secondary, n (%)	386 (21.4)	227 (24.7)	159 (18)	
Tertiary, n (%)	1418 (78.6)	692 (75.3)	726 (82)	
Public university hospital, n (%)	1439 (79.8)	743 (80.8)	696 (78.6)	0.244
Treated in specialized COPD outpatient clinic, n (%)	771 (42.9)	348 (38)	423 (48)	<0.001
Scheduled follow-up visits, n (%)				<0.001
• <6 months	748 (43.9)	233 (27.5)	515 (60.1)	
• 6–12 months	741 (43.5)	449 (52.9)	292 (34.1)	
• >12 months	216 (12.7)	166 (19.6)	50 (5.8)	
Respiratory care follow-up (years) median, (IQR)	5.2 (3.4–8.1)	4.8 (3.2–7.8)	5.7 (3.6–8.5)	<0.001

Footnote: Data presented as mean (SD) or number (percentage) or median (interquartile range); BMI: body mass index; mMRC: modified Medical Research Council; CAT: COPD Assessment Test; FEV1%: post-bronchodilator FEV1 percent predicted; KCO%: carbon monoxide diffusion factor percent predicted; BODE: body mass index, airflow obstruction, dyspnea, and exercise capacity; BODEx: body mass index, airflow obstruction, dyspnea, and severe exacerbations; GOLD: Global Initiative for Chronic Obstructive Lung Disease; GesEPOC: Spanish National Guideline for COPD; LABA: long-acting beta-2 agonists; LAMA: long-acting antimuscarinic agents; ICS: inhaled corticosteroids. Hospital complexity level II (secondary hospital): from 5 to 10 medical specialties; capacity of 200–800 beds; often referred to as a provincial hospital. Hospital complexity level III (tertiary hospital): highly specialized equipment and staff, capacity of 300–1500 beds.

**Table 2:** Factors related to clinically uncontrolled COPD. "Multivariable logistic model".

Clinical characteristics	OR	[CI 95% (OR)]	p
Age (years)	1.02	1.00–1.04	0.007
Pack-years	1.00	0.99–1.01	0.080
Charlson index <3 (ref)	1		0.067
Charlson index ≥3	1.23	0.98–1.54	
Depression, No (ref)	1		0.002
Yes	1.78	1.24–2.57	
Chronic bronchial infection, No (ref)	1		0.043
Yes	1.48	2.19	
Non-exacerbator (ref)	1		
Exacerbator with chronic bronchitis	4.87	3.06–7.74	<0.001
Exacerbator with emphysema	3.72	2.42–5.72	<0.001
Asthma-COPD	2.20	1.39–3.47	0.001
Actions	OR	[CI 95% (OR)]	p
Monotherapy (LAMA or LABA) (ref)	1		
LAMA+LABA combination	3.30	- 8.30	0.011
LABA+ICS combination	2.52	0.78–8.13	0.120
Triple therapy (LAMA+LABA+ICS)	4.08	1.68–9.87	0.002
Long-term oxygen therapy, No (ref)	1		<0.001
Yes	2.43	1.74–3.38	
Home ventilation, No (ref)	1		0.285
Yes	1.41	0.74–2.69	
Respiratory rehabilitation, No (ref)	1		0.134
Yes	1.34	0.91–1.99	
Any change in current medication advised, No (ref)	1		0.011
Yes	1.52	1.10–2.11	
Care process	OR	[CI 95% (OR)]	p
Treated in specialized COPD outpatient clinic, No (ref)	1		0.180
Yes	1.03	0.74–1.42	
Level of complexity of hospital			0.006
Secondary (ref)	1		
Tertiary	1.89	1.20–2.98	
Respiratory care follow-up (years)	0.99	0.96–1.02	0.515

Footnote: LABA: long-acting beta-2 agonists; LAMA: long-acting antimuscarinic agents; ICS: inhaled corticosteroids. Hospital complexity level II (secondary hospital): from 5 to 10 medical specialties; capacity of 200–800 beds; often referred to as a provincial hospital. Hospital complexity level III (tertiary hospital): highly specialized equipment and staff, capacity of 300–1500 beds.

### Actions taken according to control status

**Fig 3:**
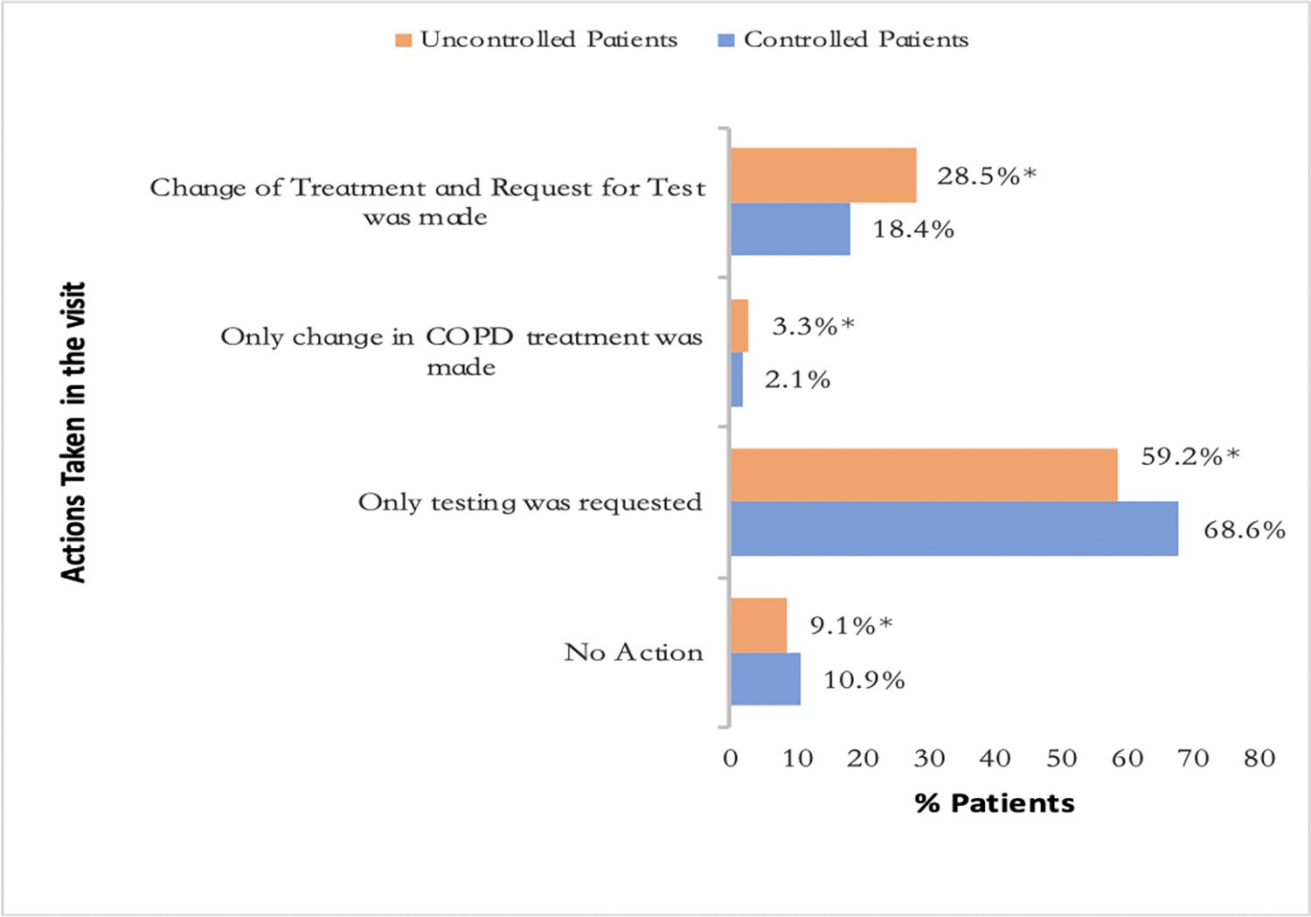
Actions taken during the visit according to the level of clinical control of COPD. Data are represented as percentages; * p<0.001.

Tables 3 and 4 and Fig 3A show the actions taken during the visit and their distribution according to the level of clinical control of COPD. Requesting a test was the most frequently performed action at the follow-up visit (87%) in controlled and uncontrolled patients, as well as inhalation technique review (65.4% in controlled and 72.3% in uncontrolled patients, p = 0.002) and adherence evaluated (56.4% in controlled and 63.2% in uncontrolled patients, p = 0.003). A change in COPD treatment was carried out during the visit in 20.3% of controlled patients versus 31.6% of uncontrolled patients (p<0.001).

**Table 3:** Actions taken during the visit according to level of clinical control of COPD.

	Controlled patients N = 919	Uncontrolled patients N = 885	p
No action, n (%)	100 (10.9)	80 (9.1)	<0.001
No change in treatment was made, n (%)	732 (79.1)	605 (68.3)	<0.001
No tests were requested, n (%)	124 (13.4)	112 (12.6)	0.674
Any change in current medication advised, n (%)	187 (20.3)	280 (31.6)	<0.001
Reason for the change, n (%)			0.057
• Level of control	83 (57.2)	149 (63.1)	
• Undesired effects	10 (6.9)	27 (11.4)	
• Compliance	18 (12.4)	29 (12.3)	
• Inhalation technique	34 (23.4)	31 (13.1)	
Change made, n (%)	187 (20.3)	280 (31.6)	
• Scaling (increased or added)	67 (7.3)	119 (13.4)	0.016
• De-escalating (decrease or remove)	23 (2.5)	23 (2.6)	0.542
• Changes to similar regimen	97 (10.6)	138 (15.5)	0.001
Request for test during the visit, n (%)	795 (87)	773 (87.6)	0.674
• Pulmonary function test	690 (75.1)	588 (66.4)	<0.001
• Imaging study	388 (42.2)	410 (46.3)	0.079
• Microbiological study	76 (8.3)	184 (20.8)	<0.001
• Blood tests	173 (18.8)	239 (27)	<0.001
• Cardiology study	38 (4.1)	64 (7.2)	0.004
Treatment adherence evaluated, n (%)	518 (56.4)	559 (63.2)	0.003
Inhalation technique evaluated in any way, n (%)	601 (65.4)	640 (72.3)	0.002
Inhalation device satisfaction evaluated, n (%)	337 (36.7)	348 (39.3)	0.246
Intervention for smoking cessation offered, n (%)	251 (27.3)	206 (23.3)	0.049
Annual influenza vaccination recorded, n (%)	842 (91.6)	820 (92.7)	0.415
Pneumococcal vaccination recorded, n (%)	760 (82.7)	751 (85.1)	0.149
Regular exercise recommended during the visit, n (%)	770 (83.8)	754 (85.2)	0.408

Footnote: Data presented as number (percentage).

**Table 4:** Actions taken during the visit according to risk level and clinical phenotype based on GesEPOC criteria.

Action taken during a visit	Low risk level (n = 419)		High risk level (n = 809)	
	Controlled patients (n = 320)	Uncontrolled patients (n = 99)	Controlled patients (n = 261)	Uncontrolled patients (n = 548)
No action, n (%)	28 (8.8)*	6 (6.1)	34 (13)*	53 (9.7)
Only testing was requested, n (%)	230 (71.9)*	64 (64.6)	162 (62.1)*	308 (56.2)
Only change in COPD treatment was made, n (%)	6 (1.9)*	0	8 (3.1)*	21 (3.8)
Change of treatment and request for testing was made, n (%)	56 (17.5)*	29 (29.3)	57 (21.8)*	166 (30.3)

*p<0.001.

### Physician’s determination of the level of COPD control and disagreement with control status

**Fig 4:**
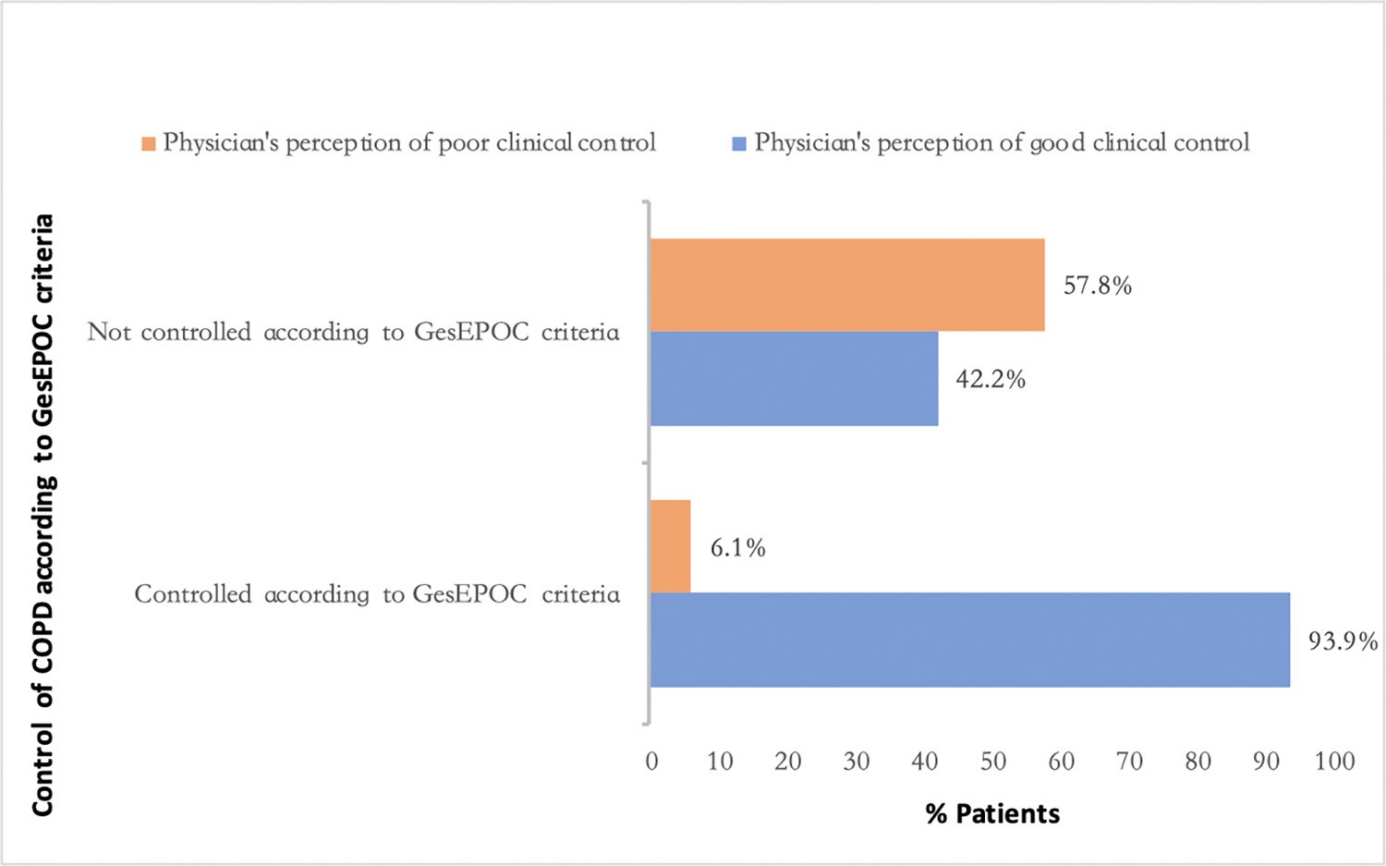
Physician’s perception on the degree control according to the level of clinical control by GesEPOC criteria. Data are represented as percentages.

Of the 1804 patients evaluated, COPD was determined to be controlled in 1102 (61.1%) cases, which was recorded at the visit by the attending physician. Fig 2B shows the distribution according to risk level and clinical phenotype based on GesEPOC criteria. Actions taken during the visit according to the physician’s classification of level of COPD control are shown in Fig 3B. In patients with uncontrolled COPD, there was disagreement with the determined degree of control in 42.2% of cases, having been reported as good clinical control. In patients with good clinical control of COPD, the attending physician reported poor control in 6.1% of patients (Fig 4). The probability of disagreement with the physician’s classification in uncontrolled patients was lower in those with severe exacerbations (OR 0.3, 95% CI 0.17–0.78, p = 0.009) and those with more exacerbations in the last year (OR 0.6, 95%CI 9.4–0.9, p = 0.019). The probability of a physician classifying control status as good in uncontrolled patients was lower if any change in current medication was advised [OR 0.27 (0.14–0.52), p<0.001] and higher when a longer check-up was scheduled. The results of the multivariable analysis are shown in Table 5. Clinical characteristics and actions taken during the visit in uncontrolled patients according to clinical determination of the degree of control are described in S1 Table.

**Table 5:** Risk factors associated with physician’s determination of good clinical control in uncontrolled patients. "Multivariable logistic model".

Clinical characteristics	OR	[CI 95% (OR)]	p
Pack-years	0.99	(0.98–1.00)	0.149
Charlson index <3 (ref)	1		0.298
Charlson index ≥3	1.36	(0.76–2.43)	
Dyspnea (mMRC) <2 (ref)	1		0.416
Dyspnea (mMRC) ≥2	0.62	(0.19–1.95)	
Post-FEV_1_% predicted	1.00	(0.98–1.02)	0.820
Number of exacerbations in the last year	0.62	(0.42–0.92)	0.019
Non-exacerbator (ref)	1		
Exacerbator with chronic bronchitis	0.32	(0.12–0.83)	0.020
Exacerbator with emphysema	0.36	(0.14–0.87)	0.024
Asthma-COPD	0.46	(0.13–1.65)	0.238
Hospital admissions in the last year <1 (ref)	1		
Hospital admissions in the last year ≥1	0.37	(0.17–0.78)	0.009
Actions	OR	[CI 95% (OR)]	p
Monotherapy (ref)	1		
LAMA+LABA combination	1.09	(0.1–10.91)	0.940
LABA+ICS combination	0.81	(0.08–8.20)	0.862
Triple therapy (LAMA+LABA+ICS)	0.71	(0.09–5.15)	0.742
Home ventilation, No (ref)	1		
Yes	0.46	(0.16–1.33)	0.155
Respiratory rehabilitation, No (ref)	1		0.099
Yes	0.54	(0.26–1.12)	
Any change in current medication advised			<0.001
No (ref)	1		
Yes	0.27	(0.14–0.52)	
Care process	OR	[CI 95% (OR)]	p
Level of complexity of hospital			0.016
Secondary (ref)	1		
Tertiary	0.22	(0.06–0.76)	
Public university hospital			0.623
No (ref)	1		
Yes	1.23	(0.52–2.90)	
Scheduled follow-up visits			<0.001
<6 months (ref)	1		
≥6 months	4.70	(2.48–8.90)	
Respiratory care follow-up (years)	0.93	(0.85–1.02)	0.147

Footnote: mMRC: modified Medical Research Council; post FEV1% predicted, post-bronchodilator FEV1 percent predicted; LABA: long-acting beta-2 agonists; LAMA: long-acting antimuscarinic agents; ICS: inhaled corticosteroids. Hospital complexity level II (secondary hospital): from 5 to 10 medical specialties; capacity of 200–800 beds; often referred to as a provincial hospital. Hospital complexity level III (tertiary hospital): highly specialized equipment and staff, capacity of 300–1500 beds.

### Clinical inertia and associated factors

There was clinical inertia in 9.1% of uncontrolled patients (Fig 3A). Factors associated with clinical inertia are shown in Table 6: anxiety (OR 2.5, 95% CI 1.1–5.7; p = 0.023), smoking habits (ex-smoker versus active smoker, OR 2.9, 95% CI 1.6–5.0; p<0.001), number of exacerbations in the previous year (OR 0.7, 95% CI 0.5–0.9; p = 0.028), and dual bronchodilator therapy (OR 0.2, 95% CI 0.05–0.93, p = 0.040). S2 Table describes the clinical characteristics of uncontrolled patients according to clinical inertia.

**Table 6:** Factors related to clinical inertia in uncontrolled patients. "Multivariable logistic model".

Clinical characteristics	OR	[CI 95% (OR)]	p
Active smoker (ref)	1		<0.001
Ex smoker	2.92	(1.68–5.09)	
Anxiety, No (ref)	1		0.023
Yes	2.54	(1.13–5.70)	
Dyspnea (MRC-m) <2, Not (ref)	1		0.073
Dyspnea (MRC-m) ≥2	2.76	(0.90–8.39)	
Chronic bronchitis criteria, No (ref)	1		0.065
Yes	0.56	(0.30–1.03)	
Chronic bronchial infection, No (ref)	1		0.326
Yes	0.61	(0.23–1.62)	
Post-FEV_1_% predicted	0.99	(0.97–1.01)	0.422
Number of exacerbations in the last year	0.71	(0.53–0.96)	0.028
Actions	OR	[CI 95% (OR)]	p
Monotherapy (LAMA or LABA)	1		
Double therapy (LAMA+LABA)	0.21	(0.05–0.93)	0.040
LABA+ICS combination	0.68	(0.16–2.86)	0.601
Triple therapy (LAMA+LABA+ICS)	0.32	(0.07–1.47)	0.146
Long-term oxygen therapy, No (ref)	1		0.567
Yes	1.21	(0.62–2.36)	
Home ventilation, No (ref)	1		0.438
Yes	0.71	(0.30–1.67)	
Respiratory rehabilitation, No (ref)	1		0.438
Yes	0.71	(0.30–1.67)	
Care process	OR	[CI 95% (OR)]	p
Public university hospital, No (ref)	1		0.211
Yes	2.14	(0.64–7.06)	
Treated in specialized COPD outpatient clinic, No (ref)	1		0.691
Yes	1.16	(0.55–2.41)	
Respiratory care follow-up (years)	1.06	(0.00–0.39)	0.007

Footnote: mMRC: modified Medical Research Council; post FEV1% predicted, post-bronchodilator FEV1 percent predicted; LABA: long-acting beta-2 agonists; LAMA: long-acting antimuscarinic agents; ICS: inhaled corticosteroids.

### Therapeutic inertia and associated factors

There was TI in 68.4% of uncontrolled patients (Fig 3A) and this was 3.3 times more likely when the attending physician reported the disease as controlled and was less likely to occur in patients with a Charlson comorbidity index ≥3 (OR 0.8, 95% CI 0.1–3.0; p = 0.014). The results of the multivariable analysis are shown in Table 7. Clinical characteristics according to TI are shown in S3 Table.

**Table 7:** Factors related to therapeutic inertia (TI) in uncontrolled patients. "Multivariable logistic model".

	OR	[CI 95% (OR)]	p
Age	1.05	(0.99–1.05)	0.058
Charlson index <3, No (ref)	1		0.014
Charlson index ≥3, Yes	0.85	(0.13–3.03)	
Chronic bronchitis criteria, No (ref)	1		0.187
Yes	0.69	(0.40–1.19)	
Chronic bronchial infection, No (ref)	1		0.185
Yes	0.68	(0.39–1.19)	
GesEPOC phenotype			
• Non-exacerbator	1		
• Exacerbator with chronic bronchitis	0.96	(0.52–1.77)	0.915
• Exacerbator with emphysema	0.71	(0.43–1.18)	0.190
• Asthma-COPD	0.97	(0.41–2.29)	0.963
Public university hospital, No (ref)	1		0.053
Yes	1.87	(0.99–3.56)	
Classified by physician as uncontrolled (ref)	1		<0.001
Classified by physician as controlled	3.37	(2.33–4.88)	

## Discussion

This study provides novel information on actions taken during the visit in COPD patients undergoing follow-up in outpatient respiratory clinics as well as factors associated with clinical inertia and disagreement with control of COPD calculated and reported by physicians using real data generated in a clinical audit performed in Spain. This analysis describes requests for testing and changes made in treatment during the visit according to the level of clinical control of COPD and explores the determinants associated with a lack of action in uncontrolled patients.

Our key takeaways are that the level of clinical control of COPD according to GesEPOC criteria was assessed in less than half of the audited visits. Clinical control in COPD is a concept that combines the impact or clinical repercussion of the disease on the patient and clinical stability (exacerbations) over time, which has been shown to be associated with better outcomes [4–7] and is recommended as a tool in the follow-up of COPD [1]. In addition, in a high proportion of COPD patients who met the criteria for poor clinical control (49.1%), no therapeutic action was taken at the visit (68.3%), with therapeutic inertia being more likely when the physician responsible for the visit reported there being good control. This study shows that the degree of control calculated and reported by the attending physician was discordant in nearly 70% of uncontrolled patients. These results suggest significant opportunities for training and improvements in the use of tools to improve COPD recognition and management.

The GesEPOC [1] recommend identifying the level of clinical control of COPD during follow-up visits to identify uncontrolled patients with greater risk of exacerbations and deterioration of their quality of life [5, 6, 17] and those that will require action to be taken. In our analysis, the least recorded criteria during the visit to assess clinical control were the use of rescue medication and regular exercise, although both are relevant data in clinical assessment, as higher use of rescue medication [18] and a low level of physical activity have been associated with worse health outcomes, such as an increased risk of future exacerbations, increased lung function impairment and risk of all-cause mortality [19, 20]. Therefore, new approaches such as the use of computer applications or scoring systems in the assessment of control [21] and the involvement of non-physician healthcare members such as nurses could facilitate assessment of control in COPD during the medical visit to provide better management of COPD, reducing clinical inertia and favoring the adequacy of treatment.

Clinical practice guidelines in COPD establish the reduction of symptoms and the minimization of the risk of COPD as the main therapeutic objectives, considering therapeutic success as the achievement of disease control. Overcoming clinical and therapeutic inertia in determining clinical control to reduce the impact and risk of exacerbations is essential to prevent further exacerbations and to reduce mortality and morbidity in people with COPD. In this context, clinical inertia is a broad concept that is often mistakenly equated with the concept of TI. Clinical inertia is defined as not acting in the case of a patient who does not achieve therapeutic objectives. Although these actions are not only focused on initiating or intensifying therapy, as there are other actions such as evaluating possible aggravating factors (requesting studies, reviewing inhalation technique, evaluating therapeutic adherence) and communication between the doctor and the patient, which will have an impact on clinical management and control of COPD. In our study, only 9% of the patients with uncontrolled COPD had clinical inertia. In the analysis of the actions carried out at the follow-up visit, the request for tests was a frequent action without differences according to the level of clinical control of COPD, although analytical, microbiological and cardiological studies were more frequent in uncontrolled patients. On the contrary, pulmonary function studies were more frequent in controlled patients. In addition, assessment of inhalation technique and adherence was more frequent in uncontrolled patients. In our study, we found a higher odds ratio for clinical inertia for factors like being an ex-smoker and having anxiety and the situation was less likely to occur in patients with higher numbers of exacerbations in the previous year and in those patients with uncontrolled disease undergoing dual bronchodilator therapy. These results may suggest that physicians do feel the need to act if the patient has a history of exacerbations and there is room for improvement in inhaled therapy. However, no association has been identified with other variables such as more significant dyspnea or a greater severity of obstruction, or treatment with triple therapy or oxygen therapy, which could reflect an attitude of acceptance and therapeutic limitation on the part of the physician. However, it should be noted that 85% of uncontrolled patients had dyspnea greater than or equal to 2 mMRC and a CAT greater than 10 and less than 20% had undergone a respiratory rehabilitation program. These findings could be partly explained by the fact that the prescription of respiratory rehabilitation is still a limited practice in many centers in Spain, where only 52.4% of the centers surveyed have a professional dedicated to offering this therapy and 38.1% of centers include less than 5 patients per month [22]. Another factor associated with clinical inertia that was identified in our analysis is the presence of anxiety in uncontrolled patients, which was present in 15% of cases. Furthermore, depression may have a negative impact on physical activity reported as a criterion defining the level of control. Previous studies have indicated that the presence of anxiety in patients with COPD is associated with increased mortality, exacerbation rates, length of hospital stay, and decreased quality of life and functional status [23].

In our study, 68.4% of the patients with uncontrolled COPD had TI, which is the failure to change therapy when treatment goals are not met, either by switching medication or increasing the dose. We found that it was 3.3 times more likely when the attending physician reported the disease as controlled. In this case, physicians do not feel the need to switch up therapies or increase doses. In our study, 42.2% of uncontrolled COPD patients were considered by physicians to be well controlled. Identifying uncontrolled patients is relevant in clinical follow-up. In our analysis, the factor with the highest odds ratio was the patient not having suffered moderate to severe exacerbations in the last year. Uncontrolled patients whose disease control was classified as good by their physician were 2.7 times less likely to receive a therapeutic action at their visit and 4.7 times more likely to be scheduled for a longer check-up, which may be a consequence or a cause. The results of the multivariable analysis suggest that disagreement between physician classification and the degree of COPD control calculated according to GesEPOC criteria is frequent in uncontrolled patients, being more frequent in patients who have a lower number or less severity of exacerbations, and this could suggest that physicians do not take further action if patients do not exacerbate, even if their COPD is considered uncontrolled due to clinical impact. The results show that physicians tend to feel more empowered to address exacerbation risk rather than uncontrolled dyspnea. Healthcare professionals must be able to recognize symptoms and exacerbations. Identifying the level of risk and the degree of clinical control of COPD is essential at the visit and is highly relevant to the measures taken, as it helps to determine the likelihood of future complications [4–6] and the appropriate management of patients with COPD. The studies that have assessed real-world prescription have shown that inappropriate therapy, undertreatment and overtreatment all lead to suboptimal management of patients with COPD. Halpin et al. [24] reported that only between 33% and 60% of patients are treated appropriately according to GOLD recommendations. Additionally, other studies have described that some patients do not receive treatment despite experiencing symptoms or having a history of exacerbations and in many cases COPD management often does not follow treatment guidelines [8, 25, 26].

Clinical inertia is broadly defined as “recognition of the problem but failure to act” [11] and includes an ample spectrum of components such as patients’ nonadherence to prescribed treatment, therapeutic inertia (providers fail to initiate medications or to intensify treatment) and inappropriate therapy, among others. The factors that may influence clinical inertia in COPD management include health professional factors and patient factors. Only 25% of care providers used the guidelines to make clinical decisions [27] and some doctors have a “nihilistic” view towards COPD [28]. Furthermore, COPD is under-acknowledged by patients when compared to other disease states such as diabetes or hypertension, and the importance of respiratory symptoms in seeking medical assistance is underestimated [29]. Many patients with COPD underestimate the severity of their disease, despite the fact that 84% of patients experienced limitations from COPD, and there is a significant disparity between self-perception and the degree of severity indicated by objective scales [30]. Therefore, the work of educating non-physician healthcare professionals is crucial. The use of work-flow strategies such as changing the responsibility of non-physicians and the use of scoring systems could help, although a multifaceted approach is required due to the myriad of factors that contribute to clinical inertia.

Our study has several limitations. The main limitation of the present study is its post-hoc design. The data presented here is the historical evaluation of a specific clinical visit. Any clinical audit has the intrinsic limitation of lost values, regardless of the inclusion methodology and periodic supervision of the database. In addition, participating centers were not selected randomly and hospitals’ participation was voluntary, depending on their previous experience with clinical studies on COPD and their interest in participating. Therefore, the results cannot be considered representative of the national population with COPD and the results of our audit could show a better picture of the care offered in centers with greater interest in and more knowledge about COPD. Another limitation to consider is that, due to the nature of the cross-sectional design, it is difficult to determine the temporal sequence when assessing factors associated with physician-determined control, as they could be either a cause or consequence. Also a limitation is the absence of information on social and educational factors of the study participants, as these are factors that may be related to disease control and inertia. Despite these limitations, we believe that this dataset represents the most extensive sample from respiratory clinics in Spain, offering real-world data on patients with COPD.

## Conclusion

This study provides information on actions taken at the visit in COPD patients undergoing follow-up in outpatient respiratory clinics and explores the determinants associated with the lack of action in uncontrolled patients using real data generated in a clinical audit performed in Spain. The results show that there is therapeutic inertia in more than half of uncontrolled patients, with this being more likely when there is disagreement with the assessment of the physician responsible for the visit who reported there being good control, this being more likely in patients with less history of exacerbations.

## Supporting information

S1 Appendix The inclusion criteria and exclusion criteria. (DOC)

S2 Appendix Risk stratification according to GesEPOC. (DOC)

S3 Appendix Clinical control of COPD according to GesEPOC criteria. (DOC)

S4 Appendix Centres and investigators participating in the EPOCONSUL 2021 study. (DOC)

S1 Table Clinical characteristics and actions taken in the visit in uncontrolled patients according physician’s determination of the level of COPD control. (DOC)

S2 Table Characteristics in uncontrolled patients according to clinical inertia. (DOC)

S3 Table Characteristics in uncontrolled patients according to therapeutic inertia (TI). (DOC)
