# Supplementary material for: Clinical control in COPD and therapeutic implications: The EPOCONSUL audit
Source: PLoS One. 2025 Jan 9;20(1):e0314299. doi: 10.1371/journal.pone.0314299 (PMC11717229; doi:10.1371/journal.pone.0314299)
Supplement: S1 Appendix — (DOC) [file pone.0314299.s001.doc]

S1 Appendix. The inclusion criteria and exclusion criteria

| The inclusion criteria | - patients aged ≥40 years  - smokers or ex-smokers (of at least 10 pack-years)  - COPD diagnosed on the basis of spiro­metric tests (FEV1/FVC post-bronchodilation<0.7 or FEV1/FVC pre-bronchodilation<0.7 and FEV1≥80%, if there is no bronchodilation reversibility testing available |
| --- | --- |
| The exclusion criteria | - lack of follow-up for at least 1 year in a respiratory outpatient clinic  - participating in a clinical trial |
