# Supplementary material for: Clinical control in COPD and therapeutic implications: The EPOCONSUL audit
Source: PLoS One. 2025 Jan 9;20(1):e0314299. doi: 10.1371/journal.pone.0314299 (PMC11717229; doi:10.1371/journal.pone.0314299)
Supplement: S2 Appendix — (DOC) [file pone.0314299.s002.doc]

S2 Appendix. Risk stratification according to GesEPOC

LOW RISK

(must meet all criteria)

HIGH RISK

(must meet at least one criterion)

Obstruction

(Post-bronchodilator FEV1%)

≥50%

<50%

Dyspnea (mMRC)

0 – 1

2 - 4

Exacerbations in the last year

0 – 1 exacerbations (without hospitalization)

≥2 exacerbations or

≥1 hospitalization
