# Supplementary material for: Clinical control in COPD and therapeutic implications: The EPOCONSUL audit
Source: PLoS One. 2025 Jan 9;20(1):e0314299. doi: 10.1371/journal.pone.0314299 (PMC11717229; doi:10.1371/journal.pone.0314299)
Supplement: S3 Appendix — (DOC) [file pone.0314299.s003.doc]

S3 Appendix. Clinical control of COPD according to GesEPOC criteria

| **Criteria for the level of clinical control of COPD** | | |
| --- | --- | --- |
| **Low clinical impact** (at least 3 of the 4 criteria must be met) | | |
|  | FEV_1_ ≥50% | FEV_1_ <50% |
| - What is the color of the sputum of the last few days? | White / clean or without sputum | White / clean or without sputum |
| - Dyspnea | 0-1 | 0-2 |
| - How many times did you use rescue medication in the last week? | < 3 times / week | < 3 times / week |
| - How much time (on average) have you walked per day in the last week? | ≥ 30 minutes per day | ≥ 30 minutes per day |
| **Clinical stability** (criteria must be met) | | |
| - Exacerbations in the last 3 months | None | |
| **Good clinical control of COPD** | Low impact + stability | |
