# Supplementary material for: Clinical control in COPD and therapeutic implications: The EPOCONSUL audit
Source: PLoS One. 2025 Jan 9;20(1):e0314299. doi: 10.1371/journal.pone.0314299 (PMC11717229; doi:10.1371/journal.pone.0314299)
Supplement: S1 Table — (DOC) [file pone.0314299.s005.doc]

S1 Table. Clinical characteristics and actions taken in the visit in uncontrolled patients according physician’s determination of the level of COPD control

| **Uncontrolled patients according to GesEPOC criteria**  N=516 | **Physician’s determination of good clinical control** (N=218) | **Physician’s determination of poor clinical control** (N=298) | p |
| --- | --- | --- | --- |
| **Clinical Characteristics** | | | |
| Gender (male), n (%) | 153 (70.2) | 219 (73.5) | 0.408 |
| Age (years), m (SD) | 71.3 (8.6) | 70.6 (9.5) | 0.377 |
| Current smokers, %  Pack-years, m (SD) | 49 (22.5)  49.0 (25.3) | 81 (27.2)  54.7 (21.2) | 0.224  0.008 |
| BMI kg/m2, m (SD) | 27.5 (5.1) | 27.3 (5.3) | 0.678 |
| Charlson index, median, IQR  Charlson index ≥3,n (%)  Obstructive apnoea síndrome, n (%)  Depressión, n (%)  Anxiety, n (%) | 2 (1-3)  59 (27.1)  59 (27.1)  37 (17)  29 (13.3) | 2 (1-3)  113 (37.9)  86 (28.9)  71 (23.8)  51 (17.1) | 0.020  0.010  0.654  0.059  0.237 |
| Dyspnea (MRC-m) ≥2, n (%) | 159 (73.3) | 275 (92.9) | <0.001 |
| CAT questionnaire > 10, n (%) | 58 (72.5) | 133 (96.4) | <0.001 |
| Chronic bronchitis criteria, n (%) | 113 (51.8) | 168 (56.4) | 0.306 |
| Chronic colonization, n (%) | 46 (21.1) | 75 (25.2) | 0.281 |
| Symptoms suggestive of asthma, n (%) | 23 (10.6) | 39 (13.1) | 0.381 |
| Post-FEV_1_, % predicted, m (SD) | 52.0 (15.9) | 45.2 (15.3) | <0.001 |
| KCO % predicted, m (SD) | 66.9 (25.7) | 61.7 (18.0) | 0.036 |
| Number exacerbations in previous year, median, IQR | 1 (0-1) | 1 (1-2) | <0.001 |
| ≥1 hospital admissions in last year, n (%) | 47 (21.6) | 181 (60.7) | <0.001 |
| BODE value, median (IQR) | 4 (2-6) | 5 (4-6) | <0.001 |
| BODEx value, median (IQR) | 3 (2-5) | 5 (4-6) | <0.001 |
| GOLD group, n (%)   - A - B - E | 21 (16.9)  60 (48.4)  43 (34.7) | 3 (1.9)  26 (16.7)  127 (81.4) | <0.001 |
| GesEPOC High risk level, n (%) | 107 (58.8) | 265 (97.1) | <0.001 |
| GesEPOC Phenotype, n (%)   - Non-exacerbator - Exacerbator with chronic bronchitis - Exacerbator with emphysema - Asthma-COPD | 96 (49)  36 (18.4)  45 (23)  19 (9.7) | 33 (11.7)  115 (40.6)  107 (37.8)  28 (9.9) | <0.001 |
| - Monotherapy (LAMA), n (%) - Monotherapy (LAMA), n (%) - LAMA+LABA combination, n (%) - LABA+ ICS combination, n (%) - Triple therapy, n (%) | 6 (2.8)  0  88 (40.9)  14 (6.5)  107 (49.8) | 3 (1)  1 (0.3)  73 (24.5)  16 (5.1)  205 (68.8) | <0.001 |
| Any change in medication advised, n (%) | 45 (20.6) | 148 (49.7) | <0.001 |
| **Referred reason** for the change, n (%)   - By level of control - By undesired effects - By compliance   By inhalation technique | 40 (88.9)  21 (52.5)  4 (10)  4 (10)  11 (27.5) | 141 (94.6)  103 (73)  9 (6.4)  15 (10.6)  14 (9.9) | 0.184  0.037 |
| **Change performed**, n (%)   - Scaling (increased or added) - De-escalate (decrease or remove) - Changes to similar regimen | 19 (8.7)  4 (1.8)  24 (11) | 74 (24.8)  8 (2.7)  71 (23.8) | 0.006  0.641  0.001 |
| Request for test in the visit, n (%)   - Pulmonary function test - Imaging study - Microbiological study - Blood tests - Cardiology study | 196 (89.9)  156 (71.6)  101 (46.3)  38 (17.4)  54 (24.8)  16 (7.3) | 274 (91.9)  198 (66.4)  160 (53.7)  91 (30.5)  98 (32.9)  35 (11.7) | 0.422  0.216  0.099  0.001  0.046  0.098 |
| Long-term oxygen therapy, n (%) | 58 (26.6) | 150 (50.3) | <0.001 |
| Home ventilation, n (%) | 18 (8.3) | 50 (16.8) | 0.005 |
| Respiratory rehabilitation, n (%) | 34 (15.6) | 71 (23.8) | 0.022 |
| **Care pathway** | | | |
| Level of complexity of hospital  Secondary, n (%)  Tertiary, n (%) | 67 (30.7)  151 (69.3) | 37 (12.4)  261 (87.6) | <0.001 |
| Public University Hospital, n (%) | 163 (74.8) | 180 (60.4) | 0.001 |
| Attended in specialized COPD outpatient clinic, n (%) | 94 (43.3) | 129 (43.4) | 0.979 |
| Scheduled follow-up visits, n (%)   - <6 months - 6- 12 months - > 12 months | 86 (40.4)  109 (51.2)  18 (8.5) | 236 (80.3)  54 (18.4)  4 (1.4) | <0.001 |
| Respiratory care follow-up (years), median (IQR) | 4.7 (3.3 – 6.7) | 5.6 (3.5 – 8.1) | 0.003 |

Footnote: Data presented as mean (SD) or number (percentage) or median (interquartile range);
