# Supplementary material for: Clinical control in COPD and therapeutic implications: The EPOCONSUL audit
Source: PLoS One. 2025 Jan 9;20(1):e0314299. doi: 10.1371/journal.pone.0314299 (PMC11717229; doi:10.1371/journal.pone.0314299)
Supplement: S2 Table — (DOC) [file pone.0314299.s006.doc]

S2 Table. Characteristics in uncontrolled patients according to clinical inertia

| **Uncontrolled patients according to GesEPOC criteria**  N=885 | **Some action was taken during a visit**  N= 802 | **No action was taken during the visit**  N=80 | **p** |
| --- | --- | --- | --- |
| **Clinical Characteristics** | | | |
| Gender (male), n (%) | 573 (71.4) | 54 (67.5) | 0.458 |
| Age (years), m (SD) |  |  |  |
| Current smokers, % | 220 (27.4) | 10 (12.5) | 0.004 |
| Charlson index, median, (IQR)  Charlson index ≥3, %  Obstructive apnoea syndrome, n (%)  Depressión, n (%)  Anxiety, n (%) | 2 (1-3)  260 (32.4)  204 (25.4)  152 (19)  113 (14.1) | 2 (1-3)  28 (35)  13 (16.3)  15 (18.8)  21 (26.3) | 0.716  0.639  0.069  0.965  0.004 |
| Dyspnea (MRC-m) ≥2, n (%) | 668 (83.9) | 74 (93.7) | 0.021 |
| CAT questionnaire > 10, n (%) | 293 (85.2) | 45 (90) | 0.361 |
| Chronic bronchitis criteria, n (%) | 411 (51.2) | 26 (32.5) | 0.001 |
| Chronic colonization, n (%) | 159 (19.8) | 8 (10) | 0.032 |
| Symptoms suggestive of asthma,n (%) | 99 (12.3) | 15 (18.8) | 0.103 |
| Post-FEV_1_, % predicted, m (SD) | 48.5 (16.6) | 44.8 (16.8) | 0.056 |
| KCO % predicted, m (SD) | 63.4 (22.4) | 57.8 (25.9) | 0.103 |
| Number of exacerbations in last year, median, IQR | 1 (0-2) | 0 (0-1) | <0.001 |
| ≥1 hospital admissions in the last year,n (%) | 298 (37.2) | 27 (33.8) | 0.547 |
| BODE value, median, (IQR) | 5 (3-6) | 6 (5-7) | <0.001 |
| BODEx value, median, (IQR) | 4 (3-5) | 5 84-6) | 0.070 |
| GOLD group, n (%)   - A - B - E | 37 (9)  130 (31.7)  243 (59.2) | 3 (7.7)  10 (25.6)  26 (66.7) | 0.597 |
| GesEPOC High risk level, n (%) | 495 (84.2) | 53 (89.8) | 0.251 |
| GesEPOC Phenotype, n (%)   - Non-exacerbator - Exacerbator with chronic bronchitis - Exacerbator with emphysema - Asthma-COPD | 201 (29.6)  195 (28.7)  204 (30)  79 (11.6) | 13 (21)  16 (25.8)  28 (45.2)  5 (8.1) | 0.093 |
| - Monotherapy (LAMA or LABA), n (%) - LAMA+LABA combination, n (%) - LABA+ ICS combination, n (%) - Triple therapy, n (%) | 14 (1.8)  231 (29)  44 (5.5)  505 (63.4) | 3 (3.8)  13 (16.7)  8 (10.3)  54 (69.2) | 0.039 |
| Long-term oxygen therapy, n (%) | 314 (39.2) | 40 (50) | 0.059 |
| Home ventilation, n (%) | 91 (11.3) | 12 (15) | 0.332 |
| Respiratory rehabilitation, n (%) | 152 (19) | 14 (17.5) | 0.751 |
| **Care pathway** | | | |
| Level of complexity of hospital, n (%)  Secondary  Tertiary | 157 (19.6)  645 (80.4) | 2 (2.5)  78 (97.5) | <0.001 |
| Public University Hospital, n (%) | 620 (77.3) | 73 (91.3) | 0.004 |
| Attended in specialized COPD outpatient clinic, n (%) | 375 (46.9) | 47 (58.8) | 0.004 |
| Scheduled follow-up visits, n (%)   - <6 months - 6- 12 months - > 12 months | 470 (59.9)  267 (34.1)  47 (6) | 45 (64.3)  23 (32.9)  2 (2.9) | 0.459 |
| Respiratory care follow-up (years), median (IQR) | 5.6 (3.6- 8.4) | 6.8 (4.0-11.5) | 0.029 |
| **Physician’s determination of the level of COPD control** | | | |
| Good control reported by the doctor, n (%) | 203 (41.6) | 15 (53.6) | 0.212 |

Footnote: Data presented as mean (SD) or number (percentage) or median (interquartile range);
