# Supplementary material for: Clinical control in COPD and therapeutic implications: The EPOCONSUL audit
Source: PLoS One. 2025 Jan 9;20(1):e0314299. doi: 10.1371/journal.pone.0314299 (PMC11717229; doi:10.1371/journal.pone.0314299)
Supplement: S3 Table — (DOC) [file pone.0314299.s007.doc]

**S3 Table. Characteristics in uncontrolled patients according to therapeutic inertia (TI)**

| **Uncontrolled patients according to GesEPOC criteria**  N=885 | **Therapeutic inertia**  (N= 605) | **No therapeutic inertia**  (N= 280) | **p** |
| --- | --- | --- | --- |
| **Clinical Characteristics** | | | |
| Gender (male), n (%) | 430 (71.1) | 198 (70.7) | 0.913 |
| Age (years), m (SD) | 71.5 (9.1) | 70.2 (8.7) | 0.037 |
| Current smokers, %  Pack-years, m (SD) | 151 (25)  53.6 (24.4) | 79 (28.2)  51.7 (22.5) | 0.304  0.257 |
| BMI kg/m2, m (SD) | 27.5 (5.5) | 27.4 (5.5) | 0.715 |
| Charlson index, median, IQR  Charlson index ≥3, %  Obstructive apnoea síndrome, n (%)  Depressión, n (%)  Anxiety, n (%) | 2 (1 – 3)  210 (34.7)  155 (25.6)  108 (17.9)  90 (14.9) | 2 (1 – 3)  79 (28.2)  64 (22.9)  59 (21.1)  45 (16.1) | 0.039  0.055  0.376  0.255  0.646 |
| Dyspnea (MRC-m) ≥2, n (%) | 502 (83.9) | 243 (86.8) | 0.274 |
| CAT questionnaire > 10, n (%) | 222 (83.8) | 116 (89.9) | 0.101 |
| Chronic bronchitis criteria, n (%) | 282 (46.6) | 155 (55.4) | 0.016 |
| Chronic colonization, n (%) | 101 (16.7) | 67 (23.9) | 0.011 |
| Symptoms suggestive of asthma,n (% ) | 76 (12.6) | 38 813.6) | 0.677 |
| Post-FEV_1_, % predicted, m (SD) | 48.5 (16.6) | 47.6 (16.7) | 0.464 |
| KCO % predicted, m (SD) | 62.8 (22.7) | 63.4 (22.9) | 0.744 |
| Number of exacerbations in last year, median, IQR | 1 (0-2) | 1 (0 - 2) | **0.651** |
| ≥1 hospital admissions in last year, n (%) | 217 (35.9) | 109 (38.9) | 0.380 |
| BODE value, median, IQR | 5 (3 – 6) | 5 (4 – 6) | 0.934 |
| BODEx value, median, IQR | 4 (2 – 6) | 4 (3 – 5) | 0.547 |
| GOLD group, n (%)   - A - B - E | 32 (10.5)  98 (32.1)  175 (57.3) | 8 (5.6)  140 (31.2)  94 (65.3) | 0.146 |
| GesEPOC High risk level, n (%) | 362 (83.6) | 187 (86.6) | 0.323 |
| GesEPOC Phenotype, %   - Non-exacerbator - Exacerbator with chronic bronchitis - Exacerbator with emphysema - Asthma-COPD | 158 (31.8)  138 (27.8)  148 (29.8)  53 (10.7) | 56 (22.8)  75 (30.5)  84 (34.1)  31 (12.6) | 0.085 |
| - Monotherapy (LAMA o LABA), n (%) - LAMA+LABA combination, n (%) - LABA+ ICS combination, n (%) - Triple therapy, n (%) | 15 (2.5)  166 (27.7)  35 (5.8)  383 (63.9) | 3 (1.1)  78 (28.1)  18 (6.5)  177 (64.1) | 0.570 |
| Long-term oxygen therapy, n (%) | 243 (40.2) | 112 (40) | 0.963 |
| Home ventilation, n (%) | 67 (11.1) | 37 (13.2) | 0.358 |
| Respiratory rehabilitation, n (%) | 117 (19.3) | 50 (17.9) | 0.600 |
| **Care pathway** | | | |
| Level of complexity of hospital, n (%)  Secondary  Tertiary, n (%) | 113 (18.7)  492 (81.3) | 46 (16.4)  234 (83.6) | 0.418 |
| Public University Hospital | 504 (83.3) | 192 (68.6) | <0.001 |
| Attended in specialized COPD outpatient clinic | 292 (48.3) | 131 (47.1) | 0.736 |
| Scheduled follow-up visits   - <6 months, % - 6- 12 months, % - > 12 months, % | 349 (59.4)  201 (34.2)  38 (6.5) | 166 (61.7)  91 (33.8)  12 (4.5) | 0.486 |
| Respiratory care follow-up (years) | 5.7 (3.7 8.4) | 5.7 (3.5 – 8.6) | 0.797 |
| **Physician’s determination of the level of COPD control** | | | |
| Good control reported by the doctor, n (%) | 173 (53.6) | 45 (23.3) | <0.001 |

Footnote: Data presented as mean (SD) or number (percentage) or median (interquartile range);
